# Evaluation of the Antioxidant Activity of Three Varieties of Honey from Different Botanical and Geographical Origins

**DOI:** 10.5539/gjhs.v4n6p191

**Published:** 2012-10-10

**Authors:** Hasan A. Alzahrani, Laïd Boukraâ, Yuva Bellik, Fatiha Abdellah, Balkees A. Bakhotmah, Sevgi Kolayli, Huseyin Sahin

**Affiliations:** 1 Mohammad Hussein Al Amoudi Chair for Diabetic Foot Research, King Abdulaziz University KAU, Jeddah, Saudi Arabia SA; 2 Department of Surgery, Faculty of Medicine, KAU, Jeddah, SA; 3 Laboratory of Research on Local Animal Products, Ibn-Khaldoun University of Tiaret, Tiaret, Algeria; 4 Department of Nutrition & Food Sciences, Art & Design College, KAU, Jeddah, SA; 5 Department of Chemistry, Faculty of Sciences, Karadeniz Technical University, Trabzon, Turkey

**Keywords:** honey, antioxidant, polyphenol

## Abstract

It is well established that honey contains substantial antioxidant compounds that could protect cell components from the harmful action of free radicals. One can speculate that these compounds may strengthen the organism defenses and consequently prevent oxidative stress in humans. Therefore, over time, impaired cells can accumulate and lead to age-related diseases. A comparative study was carried out to assess the antioxidant activity of three varieties of honey from different botanical and geographical (Manuka honey from New Zealand, Acacia Honey from Germany and Wild carrot honey from Algeria). Manuka honey had the highest phenolic content with 899.09 ± 11.75 mg gallic acid/kg. A strong correlation between the antioxidant activities of honeys and their total phenol contents has been noticed.

## 1. Introduction

Honey contains several antioxidant compounds including catalase, glucose oxidase, phenolic acids, ascorbic acid, flavonoids, carotenoid derivatives, organic acids, Maillard reaction products, amino acids and proteins (Blasa et al., 2006; Beretta et al., 2005; D’Arcy, 2005; Inoue et al., 2005; Aljadi & Kamaruddin, 2004; Fahey & Stephenson, 2002; Frankel et al., 1998). Scientists have found that oxygen may contribute to human aging and illness even it is vital to life. Cells form by-products called “free radicals” when oxygen is metabolized. Free radicals travel through the cell, disrupting the structure of other molecules and resulting in cellular damage. Such damage is believed to contribute to aging and various health problems. Humans protect themselves from the harmful effect of free radicals, partly, by taking antioxidants from high-antioxidant foods. Schramm et al. (2003) have described the effects of consuming 1.5g/kg body weight of corn syrup or buckwheat honey on the antioxidant and reducing capacities of plasma in healthy humans. They have found that plasma total-phenolic content increased as did plasma antioxidant and reducing capacities following consumption of honey. These results support the concept that phenolic antioxidants from processed honey are bioavailable, and that they increase antioxidant activity of plasma. Speculation could be made that these compounds may strengthen defenses against oxidative stress and that they might be able to protect humans from oxidative stress. Provided that the average sweetener intake by humans is estimated to be in excess of 70 kg per year, the substitution of honey in some foods for traditional sweeteners could result in an enhanced antioxidant defense system in healthy adults (Schramm et al., 2003). Beretta et al. (2005) reported the protective activity of a multifloral honey, which was standardized for total antioxidant power and analytically profiled (HPLC-MS) in antioxidants, in a cultured endothelial cell line (EA.hy926) subjected to oxidative stress. Using native honey (1% w/v pH 7.4, 10^6^ cells) showed strong quenching activity against lipophilic cumoxyl and cumoperoxyl radicals, with significant suppression/prevention of cell damage, complete inhibition of cell membrane oxidation, intracellular ROS production and recovery of intracellular GSH. Experiments with endothelial cells fortified with the isolated fraction from native honey enriched in antioxidants, exposed to peroxyl radicals from 1,1-diphenyl-2-picrylhydrazyl (AAPH, 10 mM) and to hydrogen peroxide (H_2_O_2_, 50-100 microM), indicated that phenolic acids and flavonoids were the main causes of the protective effect. Beretta et al. (2007) suggested that, through the synergistic action of its antioxidants, honey by reducing and removing ROS, may lower the risks and effects of acute and chronic free radical induced pathologies *in vivo*. The aim of this study was to evaluate the antioxidant activity of honeys from different botanical and geographical origins.

## 2. Methods

### 2.1 Honey Samples

Manuka honey (V1: *Leptospermum scoparium*) was purchased from Medihoney^®^ in UK, black forest honey (V2: *Acacia*) produced by Langanese Honig Germany and wild carrot honey (V3: *Daucus carota* L) was obtained from an Algerian beekeeper.

### 2.2 Total Phenol Contents (TPC)

We have determined the total phenol content by using a modified method of Folin–Ciocalteu as described by Beretta et al. (2005). 1 g of each sample of honey was treated with 10 ml of distilled water, mixed and filtered using a qualitative filter. 200µl of this solution was mixed with 500 µl Folin–Ciocalteu reagent (10%) for 5 min and then 1500 µl of a Na_2_CO_3_ solution were added (7.5%). All samples were incubated at room temperature in darkness for 30 min, and their absorbance was read at 765 nm. Total phenolic content was expressed as mg gallic acid equivalents (GAE)/kg of honey from a calibration curve using the equation:

y = 0.0094x+0.0299 (R^2^ = 0.998).

All samples were analyzed in triplicate.

### 2.3 FRAP Assay

The method of Yen and Duh (1993), with minor modification was used to determine the Fe^3+^ reducing power of honey. 2.5 ml of honey were combined with 2.5 ml of phosphate buffer (0.2M, pH 6.6) and 2.5 ml of 1% potassium ferricyanide. The combinations were incubated for 20 min at 50°C. After incubation, 2.5 ml of 10% trichloroacetic acid were added to the mixtures, followed by centrifugation at 3000 rpm for 10 min. The upper layer (1 ml) was mixed with 1 ml of distilled water and 0.5 ml of 0.1% ferric chloride. The absorbance of the obtained solution was measured at 700 nm.

### 2.4 DPPH Radical-scavenging Activity

Radical scavenging activity of methanolic extracts was determined spectrophometrically at 517 nm against 2, 2-diphenyl-1-picrylhydrazyl (DPPH) radical. Initially, the method was used by Blois (1958), developed and modified by Brand-Williams, Cuvelier and Berset (1995) and finally Molyneux (2004). The test is based on the color change of the DPPH solution from purple to yellow as the radical is neutralized by the antioxidants. Briefly, 0.75 mL of sample extracts were mixed with 0.75 mL of a 0.1 mM of DPPH in methanol. The measurement of radical scavenging activity was done using Trolox, BHT as standards and the values are expressed as SC_50_ (mg sample per mL), the concentration of the samples that causes 50% scavenging of DPPH radical.

### 2.5 ABTS Assay

The assay was carried out as described by Re et al. (1999). The total volume used in the original procedure was reduced to 1 ml. The stock solution, a 1:1 (v/v) mixture, of 2, 2’-azinobis (3-ethylbenzothiazoline-6-sulfonic acid) radical (ABTS) (7 mmol/l) and potassium persulfate (4.95 mmol/l), was incubated for 12 h at room temperature in dark to form radical-cation ABTS•+. The final solution was stable for at least one week at 4°C in dark. To give the absorbance values between 1.0 and 1.5 AU at 734 nm (the same absorbance value must be used for the standard and samples), the stock solution was diluted with phosphate buffer solution. The reduction of the absorbance at 734 nm was measured after 30 min (after reaching plateau). Radical scavenging activity was measured by using Trolox and BHT as standards and the values are expressed as SC_50_ (mg sample per mL), the concentration of the samples that causes 50% scavenging of ABTS radical.

## 3. Statistical Analysis

Correlations were established using Pearson’s correlation coefficient (*r*) in bivariate linear correlations (*p* < 0.01). These correlations were calculated using Microsoft office Excel 2007 and SPSS version 18.0 (SPSS Inc., Chicago, IL, USA).

## 4. Results and Discussion

The effect of honey absorption on the antioxidative capacity of plasma was tested in two studies (Schramm et al., 2003; Al-Waili, 2003). In the first study, maize syrup or buckwheat honeys with a different antioxidant capacity in a dose of 1.5 g/kg body weight were given to the trial persons. Honey caused an increase of both the antioxidant and the reducing serum capacity in comparison to the sugar control. In the second study, a diet supplemented with a daily honey serving of 1.2 g/kg body weight was given to trial persons. Honey improved the body antioxidant agents: blood vitamin C concentration by 47%, β-carotene by 3%, uric acid by 12%, and glutathione reductase by 7% (Al-Waili, 2003). It should be mentioned that the antioxidant activity depends on the botanical origin of honey (Baltrusaityte et al., 2007; Kücük et al., 2007). The antioxidant activity of honey polyphenols can be determined *in vitro* by comparing the oxygen radical absorbance capacity (ORAC) with the total phenolics concentration. There is a significant correlation between the antioxidant activity, the phenolic content of honey and the *in vitro* inhibition of the lipoprotein oxidation of human serum (Gheldof et al., 2003). Furthermore, in a lipid peroxidation model system, buckwheat honey showed a similar antioxidant activity as 1 mM of α-tocopherol (Nagai et al., 2006).

### 4.1 Phenol Content

Using the modified Folin-Ciocalteu assay, which is sensitive to phenol and polyphenol entities and other electron donating antioxidants (ascorbic acid, vitamin E), the total phenol content of the different honeys was investigated (Singleton et al., 1999; Beretta et al., 2005). The results of the total polyphenolic content for the three types of honey are represented in Table 1. This table reveals that the polyphenolic content rises according to the floral source in the following order: Manuka honey > Acacia honey > Wild carrot honey. As reported in Table 1, total phenol content was higher in all of the honeys types. Manuka honey had the highest content with 899.09 ± 11.75 mg gallic acid/kg. The content of total phenolic compounds observed in the case of Acacia honey was significantly higher than that reported elsewhere (Beretta et al., 2005; Bertoncelj et al., 2007; Piljac-Žegarac et al., 2009; Dobre et al., 2010). Manuka honey contained an elevated concentration of a trimethoxybenzoic acid and methylglyoxal; and 2-methoxybenzoic acid (Stephens et al., 2010), which could explain their significant levels of total phenol content (TPC) compared to the other honey types. The differences between honey samples, in terms of antioxidant activity, could be attributed to natural variations in composition (sugar, mineral and water content), to different location and also to different floral sources of nectar (Bertoncelj et al., 2007; Lachman et al., 2010).

**Table 1:** Phenol content (mg gallic acid/kg) FRAP values, radical scavenging activities (DPPH and ABTS) of tested honeys

Honey (100 mg/ml)	TPC (mg gallic acid/Kg)	FRAP value (ABS_700_)	DPPH (SC_50_)	ABTS^.+^ (SC_50_)
**Manuka**	899.09 ± 11.75	1.2106 ± 0.005	13.46±0.072	43.25±0.681
**Acacia**	627.56 ± 44.03	1.366 ± 0.06	13.62±0.054	44.37±0.790
**Wild carrot**	503.09 ± 8.29	0.6386 ± 0.05	53.31±0.084	202.26±1.033
**Trolox**	-	-	0.004±0.001	0,083±0.005
**BHT**	-	-	0.013±0.001	0,181±0.009

### 4.2 Ferric Reducing Antioxidant Power (FRAP)

The FRAP assay is the only one that directly estimates antioxidants or reductones in a sample, and is based on the ability of the analyte to reduce the Fe^3+^/Fe^2+^ couple (Lim & Tee, 2007). It has been found that the total polyphenolic content and the Fe^2+^ content formed in the presence of the honey antioxidants are significantly correlated. Similar findings were reported by others (Bertoncelj et al., 2007; Blasa et al., 2006; Beretta et al., 2005; Aljadi & Kamaruddin, 2004). In fact, all the honey samples from different sources exhibited the reducing power. However, the values in table 1 show that Acacia honey is slightly higher than that of Manuka honey, while Wild carrot seems to exert the lowest reducing power. The reducing properties are generally related to the presence of reductones (Duh, 1998). It has been stated that the antioxidant activity of reductone was based on the breaking of the free radical chain by donating a hydrogen atom (Gordon, 1990). Reductones also react with certain precursors of peroxide, thus preventing peroxide formation.

### 4.3 DPPH Radical-scavenging Activity

According to Yamaguchi et al. (1998) and Lo Scalzo (2008), DPPH analysis is one of the tests used to prove the ability of antioxidant components to act as donors of hydrogen atoms. It has been recognized that the bleaching of the DPPH solution increased regularly with increasing amount of polyphenols (Lim & Tee, 2007). Table 1 show the scavenging ability expressed as SC_50_ on the DPPH radical. Similarly, a high correlation between phenols and DPPH activity was observed indicating that phenolics chemicals govern the antiradical potency and are responsible for the antioxidant effects (*r* value was 0.9079 at P<0.05). These findings are in agreement with those reported by Beretta et al. (2005) and Dobre et al. (2010), who found a high correlation between the total antioxidant activities of various honey and their total phenol contents. However, the studied honeys showed less antioxidant activity than the standards. Therefore, the scavenging effects observed are classified in the following decreasing order: Trolox > BHT > Manuka honey > Acacia honey > Wild carrot honey.

### 4.4 Antioxidant Activities Estimated by ABTS Assay

The ABTS assay is one of the most frequently used analytical strategies for antioxidant activity. A good correlation is noticed between this assay and other methods such as the 2, 2’-diphenyll- picrylhydrazyl assay (DPPH), and the ferric reducing antioxidant power assay (FRAP). The reliable method to determine radical scavenging capacities implicates the measurement of the vanishing of free radicals, such as the 2,2´-azino-bis (3-ethylbenzenthiazoline-6-sulphonic) acid radical (ABTS•+), the 2,2-diphenyl-1-picrylhydrazyl radical (DPPH•+), or other colored radicals, with a spectrophotometer (Miller et al., 1993). The same results observed in the case of DPPH assay is observed in the ABTS method. The sources of analysed honey samples as well as floral origin differed to a large extent, as shown in Table 1. The observation that the mean SC_50_ values determined in the ABTS assay is significantly higher than the mean SC_50_ determined in the DPPH assay is explained by the fact that DPPH· radical reacts only with lipophilic antioxidants while ABTS·+ radical reacts with both hydrophilic and lipophilic antioxidants (Prior et al., 2005). Considering the varying reaction conditions employed by different authors, it is difficult to make direct comparisons of radical scavenging capacities of honeys with available literature data. The explanations behind the evidently higher radical scavenging capacity, as well as ferric reducing antioxidant power, exhibited by honeys, mainly Manuka honey, probably lie in their diverse botanical and geographical origins. Phenolic compounds play a major role in the antioxidant activity of natural products and the differences between honey samples in terms of antibacterial and antioxidant activity could be attributed to the natural variations in floral sources of nectar and the different locations. Although honey by itself may not serve as a major source of dietary antioxidants, it demonstrates the potential to play a role in providing antioxidants in a highly palatable form. Due to honey’s pleasing taste, it may be more readily consumed by individuals reluctant to ingest plant-derived antioxidants.
